# Nashville the Mecca for 1897

**Published:** 1897-03

**Authors:** 


### NASHVILLE THE MECCA FOR 1897.

MUCH interest has centred in the choice of the Executive Committee of the U. S. V. M. A. in selecting one from the many places suggested for our pilgrimage in 1897. The keenest desire has been shown by the veterinarians of the South that the meeting should be held in Nashville, where for the first time our Association will gather on Southern soil. Many were the appeals and letters received by the members of the Executive Committee commending the rival cities, but none were so forcibly presented as those which emanated from the South.

The Centennial Exposition to be held in Nashville during the coming summer, and which will close in October, offered inducements of no little import to the members of the profession in cheaper fares; and the attraction of the exposition and the open doors of hospitality which will swing inward for 1897 for all who may journey to that city seemed to weigh with the Executive Committee, so that for first choice Nashville received ten votes of the twelve votes cast; the two additional ones were for Nashville as second choice. This convention will create a new era in the South, and its members can well journey to the "Athens of America for '97," and there, in sight of the "Acropolis," reared in all its ancient form and beauty, they can seriously deliberate over the many matters of peculiar interest to us as a profession and of profound interest to our country as a race of people, particularly in the line of sanitary questions that are now attracting the attention of the learned people of the entire earth.

In the consummation of this meeting in its fullest and most complete worth for the profession there will be a work for every member to do in contributing to its success. While the warmest and most open-hearted hospitality will no doubt be tendered us in that full measure for which the Southern people are famous, it must be looked forward to not alone as a pleasure in this direction, but that there is a great work to accomplish, and it must be done thoroughly and conscientiously, in order that it will leave its impress in the South toward creating there a higher appreciation of the veterinary profession and to broaden that field for the employment of a much larger number of our representatives. It must do there as it has done in other sections of our country—stimulate and increase local and State association interests and awaken the people to the needs of wise veterinary sanitary police measures; it must also impress upon them the fact that such measures can only emanate from well and thoroughly educated members of the profession, and that these can only come from institutions of learning where a rigid and high standard of education is maintained.

Let everyone enter upon this work with a determination to do well his part, and our watchword of "On to Nashville" for 1897 will be full of meaning and freighted with responsibility to be discharged with the utmost fidelity to the interest of one and all that the members of the profession have accepted in becoming its devotees.

